# *SCL19A3* gene mutation with Leigh-like phenotype presentation: a potentially treatable disease

**DOI:** 10.1055/s-0043-1772606

**Published:** 2023-10-13

**Authors:** Leonardo Furtado Freitas, Eduardo Carvalho Miranda, Thelma Ribeiro Noce, Aline Pimentel Amaro, Márcio Luís Duarte

**Affiliations:** 1 McGill University, Department of Radiology, Division of Neuroradiology, Montreal QC, Canada.; 2 Rede MaterDei de Saúde, Departamento de Neurorradiologia, Belo Horizonte MG, Brazil.; 3 Rede MaterDei de Saúde, Departamento de Neurologia Infantil, Belo Horizonte MG, Brazil.; 4 Hospital Felício Rocho, Belo Horizonte MG, Brazil.; 5 Universidade de Ribeirão Preto, Campus Guarujá, Guarujá SP, Brazil.

**Figure 1:**
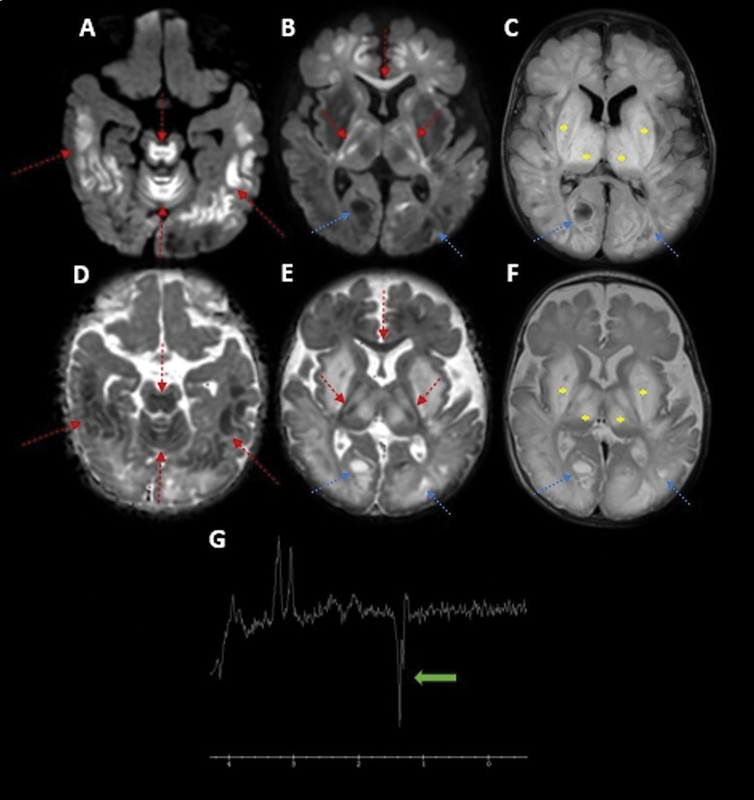
Brain magnetic resonance imaging (MRI) scan at two months of age. Axial diffusion ( **A** , **B** ); fluid-attenuated inversion recovery (FLAIR) ( **C** ); apparent diffusion coefficient (ADC) map ( **D** , **E** ); and T2-weighted imaging ( **F** ). Symmetric areas of cytotoxic (red arrows) and vasogenic (asterisk) edema in the midbrain, superior vermis, subcortical white matter of the cerebral hemispheres, genu of the corpus callosum, internal capsules, and basal ganglia, mainly in the putamina and thalami. There are also some cystic changes in the occipital lobes (blue arrows). Magnetic resonance spectroscopy (MRS) ( **G** ) with intermediate TE (144 ms) showing the lactate inverted peaks at 1.3 ppm chemical shift (green arrow).

A two-month-old boy presented with breastfeeding difficulty, hypoactivity, hyporeactivity, and seizures. Neuroimaging showed multiple areas of cytotoxic and vasogenic edema in the midbrain, cerebellum, basal ganglia, and brain hemispheres, with lactate peak ( Figure 1 ). Exome sequencing revealed a heterozygous mutation c.597dup (p.His200Serfs*25) in the *SCL19A3* gene.

**Figure 2:**
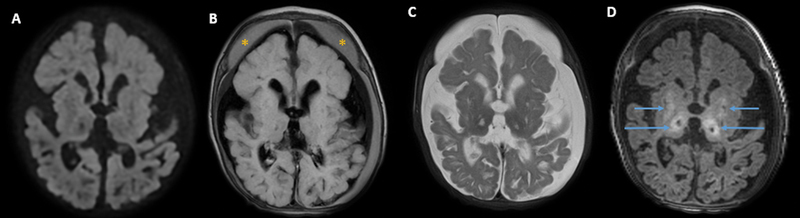
Two-month follow-up after treatment with biotin and thiamine. Axial diffusion ( **A** ); FLAIR ( **B** ); T2- ( **C** ) and T1-weighted ( **D** ) multiplanar gradient-recalled (MPGR) imaging. Resolution of the areas with restriction diffusion and interval encephalomalacia and necrosis in the basal ganglia bilaterally, mainly in the thalami (arrows). There is diffuse cerebral atrophy and holohemispheric subdural hematomas (asterisk), due to stretching of the bridging veins.

The *SCL19A3* gene encodes thiamine transporter-2. Mutations can result in thiamine metabolism dysfunction syndrome-2, and they can present as early as infantile Leigh-like syndrome, 1 a potentially treatable disease. This patient was submitted to thiamine and biotin replacement, but his evolution confirmed a poor prognosis ( Figure 2 ). 2 The poor outcome warrants genetic counseling for the families.
